# Phenomenal interface theory: a model for basal consciousness

**DOI:** 10.1098/rstb.2024.0301

**Published:** 2025-11-13

**Authors:** Colin Klein, Andrew B. Barron

**Affiliations:** ^1^ School of Philosophy, Australian National University, Canberra, Australian Capital Territory, Australia; ^2^ School of Natural Sciences, Macquarie University, Sydney, New South Wales, Australia

**Keywords:** insect, sentience, mushroom body, motivation, central complex

## Abstract

An increasing number of authors are willing to attribute phenomenal consciousness to relatively simple organisms like insects. Yet it is not at all clear what functional role the substrates of consciousness would play. Here, we argue phenomenal consciousness is a consequence of how mobile animals with spatial senses and a capacity for goal-directed behaviour resolve the complex problem of action selection. To adjudicate between possible goals an animal must use sensory inputs, representations of internal state and stored knowledge of values to estimate expected value vectors for different options. Brains solve this problem by taking such heterogenous information and transforming it into a common framework—a phenomenal interface—and then using this to compute multi-objective *Q*-values. We use insects to flesh out the details of the phenomenal interface. A consequence of this type of processing is that it naturally generates a distinction between self and non-self and a first-person perspective in which external stimuli have a subjective value. We discuss the consequences of this theory for understanding the evolution and distribution of phenomenal consciousness and suggest an underappreciated problem that arises when thinking about how consciousness might have expanded and changed as it evolved from its simplest origins.

This article is part of the theme issue ‘Evolutionary functions of consciousness’.

## 1. Introduction

Which animals are conscious? Scientific consensus is changing. In 2012, the influential Cambridge Declaration on Consciousness [1] recognized consciousness in all mammals and birds and entertained the possibility for ‘many other creatures, including octopuses’. The recent New York Declaration on Animal Consciousness extended the realistic possibility of consciousness to all vertebrates and many invertebrates ‘including, at minimum, cephalopod molluscs, decapod crustaceans and insects’.

The latest declaration relies heavily on a weight-of-evidence approach, gathering proposed behavioural markers of conscious experience in animals. This ‘theory neutral’ [2] approach is appropriate if the objective is to assess whether a certain animal group is conscious. Yet even if we conclude that insects are likely capable of conscious experience, we might still wonder *how* and *why* they have this capacity. While we agree with Birch [2] in that one of the challenges for consciousness research is that we have no consensus theory of consciousness, we think that without such a story a theory-neutral approach will always feel wanting [3].

The demand for theory becomes more challenging as the attribution of consciousness grows broader. The hard bodies of invertebrates and the differing demands of their short lifecycles mean that interpreting behavioural signs of consciousness can be fraught. The brains of most invertebrates are minuscule compared with those of birds and mammals and are arranged very differently. Hence, there is a need for a theory that gives some plausible mechanisms for consciousness.

We are concerned in particular with the most basal forms of consciousness: that is, on whether an animal has any conscious mental states at all. Human consciousness is a complex phenomenon [4], and we exclude the more complex phenomenon for discussion. The question can be asked in many ways; for present purposes, we will consider these to be synonymous. So, one might ask whether an animal is aware of things [5]. Or as Nagel [6] puts it, whether ‘there is something that it is like to *be* that organism—something it is like *for* that organism’. We assume that there is something it is like to be a bat, and nothing it is like to be a rock. Some use the term *sentience* [7] or *phenomenal* consciousness (where the latter is contrasted with phenomena like *access* consciousness or self-awareness) [8].

We have argued all vertebrates and some invertebrates likely possess a basal form of consciousness [9,10], and this view is less extreme than it once was. Of course, if you come from a very different starting point—if, for example, you think that the sophisticated higher order cognition unique to humans or neocortical processing is also a precondition for phenomenal consciousness—then a project like ours will have little appeal. Most theories of phenomenal consciousness in humans either tacitly [11,12] or explicitly [13,14] appeal to cortical mechanisms. These theories obviously cannot be extended to invertebrates. Some authors would argue that most animals lacking a human neocortex, or something like it, are capable of perception, but not awareness of what they are perceiving (a state described as transitive creature consciousness) [14]. We, however, are persuaded by arguments that a cortex is not a prerequisite for all forms of conscious experience. Bjorn Merker developed a subcortical theory of phenomenal consciousness [15,16]. He also recognized that the neural systems necessary for phenomenal consciousness have homologs in all vertebrates and consequently argued that consciousness is present throughout vertebrata [15]. Elsewhere, we extended Merker’s argument even further by demonstrating that at least analogues of the key neural systems emphasized by Merker occur also in arthropods [9,10].

Our earlier work relied on a loose generalization of Merker’s claims, but a full defence requires further abstraction. Recent advances in insect neuroscience now let us move beyond analogies and to generalize subcortical theories. Here we argue that, at a high level of abstraction, insects and vertebrates possess a similar behavioural control system that interfaces sensory information and interoceptive information into a common referential framework. We call this a *phenomenal interface* (PI). Its function is to solve problems common to mobile animals: resolving competing behavioural priorities, selection of a single appropriate action and compensating sensory input for the liabilities of self motion. An outcome of performing these functions is that a PI processes its sensorium from an egocentric and subjective perspective. This gives both a general theory of consciousness and a specific story about where to look to flesh out the details.

It is important to be clear about the scope of our strategy. We begin by *assuming* that activity in certain kinds of structure is sufficient for consciousness. Our goal is to characterize that process in a suitably abstract manner. Structural similarities between conscious experience and the computational processes so characterized are evidence that we have got things correct. What we are very much *not* doing is arguing from first principles that a certain type of computation is identical to the bases for conscious experience. To demand that would be to demand we solve the hard problem—that s a tall order.

One might instead view the strategy as analogous to Morgan’s early work [17] explaining gene linkage via chromosomal position. This required the (reasonable) assumption that chromosomes had something to do with inheritance, and that the physical processes involved in meiosis had effects on the bases of genes. Armed with that, Morgan could give a good characterization of the mechanisms of linkage. He could do so *without* knowing how chromosomal material was the basis of heredity. Similarly, our goal is to give a plausible theory about the processes that support subjective experience. Like Morgan, we think we can do so in the absence of a full story, and that doing so is a step along the way to telling a full story.

## 2. The core problem

Any mobile animal with a reasonably complex body and sensorium faces a difficult decision-making problem. Consider the task faced by a foraging bee. First, the bee has multiple needs and corresponding sources of resources: she must choose between nectar and pollen, she must balance the energetic demands of foraging and decide when to return home and she must do so while avoiding predators and dangerous obstacles. The value of different actions is context-dependent: different foods have different utility when she is personally hungry or when her colony needs specific nutrition.

While very simple organisms may get by with a fixed lexical ordering over priorities [18], a fixed ordering lacks the flexibility needed to accommodate multiple context-sensitive demands [19,20]. Nor is it clear that multiple sources of reward can or should be linearly combined into a single scalar value [21,22]. The bee also faces a classic problem of sparse reward [23]: successful foraging only pays off upon return to the hive, which means that proper behaviour must be shaped in the absence of immediate reward. Finally, the bee faces a complex sequential decision-making task: each action affects the ability to perform future actions, both in terms of where the bee ends up and simply because foraging for food itself takes energy [24,25].

The task faced by the bee is thus best modelled as a nonlinear multi-objective Markov Decision Process (nl-MOMDP) [26,27]. nl-MOMDPs are widely recognized in the reinforcement literature as particularly difficult to solve [26,27], and may admit of no computationally tractable exact solutions. Nevertheless, the bee does solve it.

The underlying computational problem required to solve the nl-MOMDP task is threefold. First, the bee must use sensory inputs, representations of internal state and stored knowledge of value to output pairs of actions and corresponding expected value vectors (*Q*-values [22,28]). Second, the bee must adjudicate between *Q*-values in order to pick a single action to perform (or continue performing). Third, upon executing an action, the bee must update her estimates of internal state and the value of different external states using any obtained reward.

**Figure 1.**
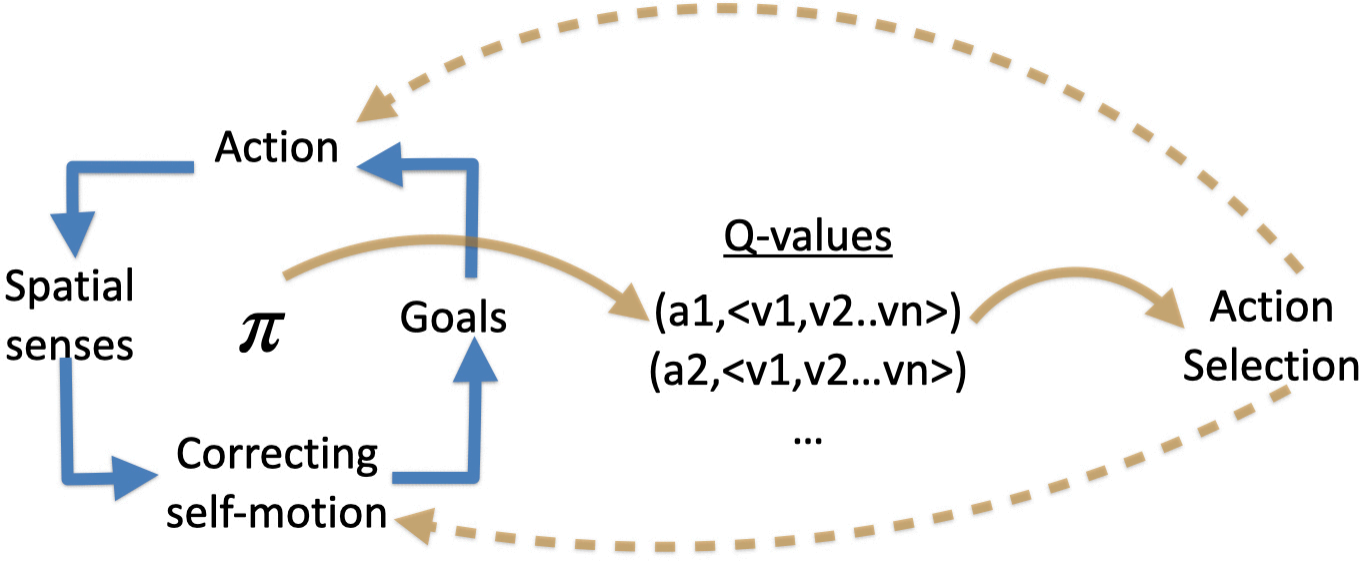
A schema of the computation performed by a phenomenal interface. (Left) The four domains that must be integrated. Blue arrows mean ‘does not make sense without’. The phenomenal interface, π, must provide a simultaneous solution. (Centre) The output of the phenomenal interface is a set of pairs of action and value-vectors. (Right) Action selection adjudicates between *Q*-values. Action selection is downstream of phenomenal consciousness but provides feedback required both for updating action values and for correction of self-motion.

The focus of phenomenal interface theory (PIT) is on the first of these tasks. We suggest that the computational function of a phenomenal interface is, first and foremost, to take pre-processed sensory input and use that to estimate the value of actions (in the sense of expected reward). Doing so requires solving a series of interconnected problems. The particular computation involved must be sensitive to each of these, and we suggest that the biological function of a PI is to solve each efficiently by doing so simultaneously (figure 1). We call it an *interface* to emphasize the role it plays in bringing diverse sources of information together, and we claim that it is *phenomenal* because it has the right structural features to underlie subjective experience. Both of these claims will be elaborated as we go.

The computational problem solved by an interface has a number of aspects. Sensory input cannot be meaningfully interpreted without correcting for self-motion [29–32]: an insect is both moving through the world and also moved by it (such as by gusts of wind), and parsing out the difference is crucial [33]. Different sensory systems and the motor system itself use fundamentally different coordinate systems that require nontrivial alignment [34]. Determining the value of actions requires combining information about current state and goals with what is perceived [35,36]. The functional role of a PI is to take this heterogenous information and transform it into a common framework and then use this to compute multi-objective *Q*-values.

A crucial feature of PIT is that this common framework need not be either pre-determined or simple. One need not assume (as Merker sometimes suggests) that the representation takes the form of a simple map of the organism in the world. The representational basis of the common framework is functionally specified and takes whatever form is necessary to most effectively solve the core problems [37]. The function of a PI is in part to *create* a common representational framework within which *Q*-values can be determined.

It is important that the process of creating such a framework is flexible over both developmental and evolutionary timescales. The particular details of the PI for a bee and ant will be very different, because their abilities and needs differ. But they can share the same computational mechanism. We envision a phenomenal interface as a classifier that can be described at a relatively high level of abstraction, but which primarily learns the details of a specific animal’s body, needs and capacities in order to generate appropriate *Q*-values.

The downstream adjudication between *Q*-values is beyond the scope of PIT (aside from the assertion that there should not be a simple linear mapping to a single scalar value). We think it can be done in a variety of context-sensitive ways [21]. There is a large literature on dealing with multi-objective decision problems [38]. Some of this will be heuristic (e.g. continuing action unless there is compelling reason to switch). Different animals might use different heuristics and decision heuristics might depend on, e.g. mood or overall arousal. The PI makes a variety of strategies possible but does not demand one; the point is that none of this adjudication can be until one can calculate *Q*-values in the first place, and doing *that* requires a common representational framework that spans world, value and action. Similarly, learning and updating of stored value are outside the scope of PIT, but the problem of sparse reward and credit assignment is facilitated by representation of actions in a common framework.

## 3. Phenomenal interface theory proper

The system we call the PI solves the basic action selection problem for complex mobile animals. Why think, however, that this has anything to do with subjective experience? We suggest that possession of a PI naturally gives rise to key features of subjectivity in particular: a first-person perspective in which stimuli have a subjective value. We flesh this out by reference to the insect central complex and associated systems, but we emphasize [10] that one finds similar integrated systems, playing a similar role, across phyla, especially vertebrate subcortical systems [16,39]. While we do not reiterate the argument here, arguments for the subcortical basis of subjective experience in humans [16,40–43] provide important convergent evidence for the role of a PI in subjective experience [9,16].

In the insect brain, there are just two multisensory integration regions: the central complex and the mushroom bodies (MB). These operate together as a system of systems to constitute the insect PI. The ancestral function of the insect central complex (CX) is correcting for the effect of self-motion in order to hold a heading towards a goal [44,45]. Insects are small and easily moved by the medium in which they travel. Without correction for self-motion, information about the world becomes noisy and temporally smeared [46–48], limiting the possibilities for effective target acquisition and navigation. The central complex is thus crucial for these functions. Insects can effectively factor out the consequences of self-motion generated by turning from their visual input using an efference copy of their predicted motor displacement to cancel out the visual consequences of the turn [49]. Note that this is partly a function of how self-movement disrupts sensory inputs and the consequences of such disruption for action: chemotaxis along a gradient (as found in bacteria and simple worms) may not need much correction for self-movement, but image-forming eyes are useless without it [48,50].

Processing within the central complex enables an insect to establish and hold a heading relative to an external navigational reference [45,51]. The neural representation of that heading can be maintained even if the fix on the external reference is temporarily lost [51,52], which enables insects to maintain a heading and to store vectors of travelled paths in memory [53]. This shows at least an elementary form of spatial representation and forward modelling of behaviour and its outcomes.

We suggest that the ability to correct for self-motion in order to hold a heading, when done consistently, can form the basis of a representation. As Poincaré [54,55] argued, the algebraic structure determined by the basic operations of self-correction is sufficient to define a geometry with an origin [54]. We will not make assumptions in what follows about the geometry of the space so defined; as Poincaré and later authors [56–58] noted, the geometry so recovered might be Euclidean, affine or other possibilities. However, we will assume that the geometry is enough to support a perspective structure.

The relationship between self-motion and passive changes in environment allows the animal to triangulate its location in a world that contains both perception and action [59]. The properties of distal senses provide further perspective structure when combined with self-motion [47,60,61]. Visual objects occlude one another in ways systematically related to motion [34,62]. In insects, phenomena like optic flow [46,63] and visual parallax [60,64,65] partition the moving world into near and far [66]. Each of these depend constitutively on the ability to distinguish self-motion via action [64,66], giving what is known as an agentive view of self-location and perspective [34].

We stress that this perspective structure remains lightweight and tacit. A need not give a full-fledged representation of oneself as a self—does not, in philosophical terms, provide a *de se* representation [67]. Instead, following Schellenberg, a PI gives a *de hinc* representation: a representation of the world ‘from here’ [68]. A *de hinc* representation is arguably the minimal way in which a conscious animal can represent its world.

In addition to appropriate spatial structure, a phenomenal interface also attaches subjective value to stimuli, so that stimuli become attractive or aversive or otherwise valenced. In insects, small populations of aminergic cells signal aspects of the state of the insect and also provide reinforcement signals to the learning and memory centres of the mushroom bodies [69,70], so that the subjective meaning of objects in the environment to the organism can be learnt [70–72]. The mushroom bodies and the CX work together as a system such that what has been learnt about an object (via mushroom body output circuits) will influence whether the insect orients toward or away from an object (via the spatially structured representation of the object in the CX) [73].

The perceived valence of an object depends not only on past encounters with a stimulus but also the present physiological state of the insect. For example, other aminergic systems associated with the mushroom bodies modify the neural output of the mushroom such that food-associated stimuli are only attractive if an insect is food-deprived [74]. Further specific aminergic circuits will activate the mushroom body outputs when entering environments associated with feeding [75], but flies can only learn about food stimuli if they are hungry [69].

Taken together, we see in insects a system of systems involving the mushroom bodies, the CX and associated neuromodulatory networks that attach subjective value to stimuli and relate those values to physiological or subjective state. This provides a mechanism by which the world may be given meaning relative to the needs of the organism.

It is well established that Hebbian-like plasticity in connections at the output of the mushroom body can support various forms of reinforcement learning [71,76–78]. More recent work [23] exploring the theoretical capacities of gap junctions in the mushroom body network suggests the mushroom bodies could have the capacity to solve the so-called ‘sparse reward’ problem: this being that in nature the actions needed to obtain a reward may be long and convoluted, with actual reward obtained only rarely. The behaviour of foraging honey bees demonstrates they can overcome this problem. Bees connect up long foraging flights and specific flower-handing strategies to the eventual consummatory act of obtaining a floral reward. Experience-dependent modulation of gap junctions connecting the neurons of the mushroom bodies could support the mapping of states to outcomes such that a sequence of actions can be learned when the reward is obtained only on completion of the sequence [23]. In this way, the mushroom bodies could support the mapping of objectives to actions and targets, even if the actions involved are a complex sequence.

Finally, the mechanisms that support reinforcement learning and motion correction in insects appear to be sophisticated enough to allow forward modelling and prediction of the behaviour of the world, including of other entities. For example, neurons in the dragonfly visual lobes sensitive specifically to small moving targets show a modulated gain of function that predicts the anticipated position of the target based on its current trajectory [79]. Together these capacities to connect sequences of events to objectives, maintain headings and forward-model actions enable insects to robustly map functional connections between objectives and targets and to maintain and execute courses of actions to achieve those targets. This could enable ants to successfully reorientate having been blown away by a gust of wind [33], or construct complex routes or new motor sequences, such as has been documented in bees, spiders and grasshoppers [80].

**Figure 2.**
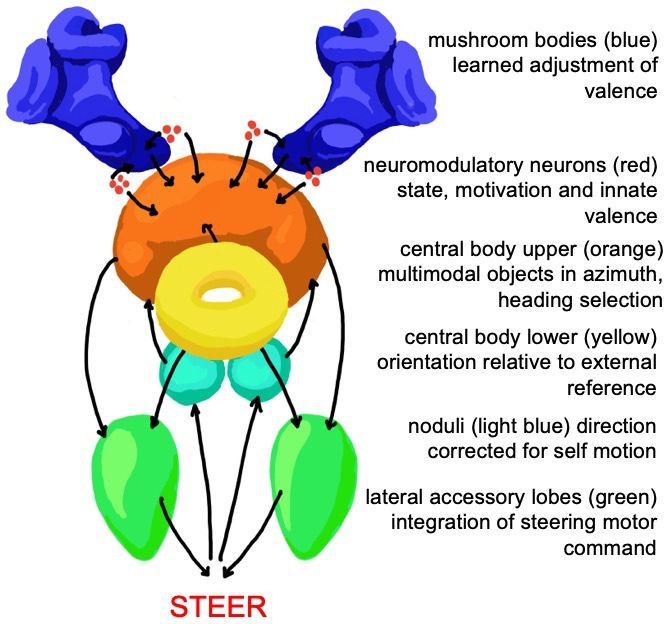
Simplified schematic of the functions and connections between the multimodal integration centres of the insect brain. Shown (not to scale) are the mushroom bodies, neuropil of the central complex and the lateral accessory lobes. Arrows show information flow. The output of these interacting systems is a steering command determined relative to subjectively weighted objects in the environment.

In summary, we see in the processing performed by the insect brain the type of structural features that matter for an animal to have a subjective experience (figure 2). The insect brain establishes a first-person perspective on the world. This differentiates self from non-self (in a simple but fundamental way), and the external world is represented with sufficient coherence, meaning and structure that the insect can operate with agency within that world. It can effectively assess the world around it and accordingly plan toward achieving personal goals. That planning is enabled because stimuli and objects in the world carry an entirely subjective weighting or valence, which is moderated by the unique state and prior experience of the insect. Hence, how any insect perceives the world will be unique to that individual insect.

The insect example has let us ground PIT in biological reality, but abstracting away shows why the *type* of processing performed by a phenomenal interface is a good candidate for an abstract theory of the mechanisms for phenomenal consciousness. This example also illustrates that a claim of phenomenal consciousness does not require an animal producing a rich veridical three-dimensional representation of the world in its head, within which it moves about to plan actions. Phenomenal consciousness is not about the richness of mental imagery. It is about representing a structured version of the world that is interfaced with and influenced by the animal’s subjective state.

## 4. Phenomenal interface theory as a neurocomputational framework

We have described the phenomenal interface at a computational level [81] and suggested reasons to think that it has the right sort of properties to support the basic capacity for subjective experience. PIT itself, however, is not a fully computational theory of consciousness. Instead, we take PIT to be a form of what philosophers call *realizer functionalism*: PIT specifies the role that an interface plays, but the claim is about an interface as instantiated in biological systems [82].

The basis of conscious experience thus relies on a particular, as yet unknown, transformation function—call it π—which serves as a phenomenal interface. Variation among individuals and species means that π itself must be understood at a suitably abstract level. Visual input is an important modality in our insect example of π. But the visually impaired are conscious too, and their blindness will give rise to a different way of solving the transformation problem, which means the particulars of their phenomenal interface will be different.

This distinction between the computational gloss and the details of π is important for two reasons. First, it re-emphasizes that π solves a particular kind of problem *in an evolvable way*. We have proposed the development of a PI is an evolvable solution to the core problem of behavioural control for mobile animals. For example, the central complex is extremely well-conserved across insect lineages and features in other arthropods too [83]. This suggests that the computation it performs is relatively agnostic to the sorts of interoceptive information available. The right level at which to understand π, then, is one on which it can function as a computational invariant across very different biologies—and can therefore integrate information as a system evolves. The possession of a phenomenal interface allows the problems identified above to be efficiently solved even as phenotypes change in drastic ways. Incorporating a new sense organ, or a new pair of appendages, does not require solving the same problems from scratch.

What is fed into a PI need not be just more elaborate sensory information but can also include arbitrarily processed information as well. Merker [16,39,84] recognized a version of a PI instantiated by subcortical structures in the mammalian brain. In the typical mammalian brain, however, recurrent loops between the subcortex and regions of the cortex are a vital and almost universal feature of processing [85]. Merker [16,39,84] has emphasized subcortical structures in the mammalian brain PI, but via these recurrent loops the PI is connected to the cortex, and the cortex adds input to the mammalian PI. The cortex would add to mammalian conscious experience modelling of the consequences of action that have greater spatial or temporal extent: projecting models of the consequences of actions across larger associative spaces. The output of this modelling returns as an input to the PI to refine action selection.

This means, note, that in more complex brains, there will be portions of cortex that perform computations that are similar to PIT, or which handle PIT-like functions. These are not necessarily conscious on their own: cortical information becomes conscious processing only insofar as it is able to enter as input into the more basic structures that compute π.

This fits well with the sort of model elaborated by, for example, Cisek [86], on which control of behaviour was shifted to the forebrain in early mammals, allowing for an elaboration of the behavioural repertoire. On the sort of model that Cisek [86] has proposed, older circuits like the basal ganglia (which we identify as part of the basis of computing π) remain responsible for between-action adjudication and the general problems of learning and resolving nl-MOMDP problems, but cortex can learn to detect new opportunities for action and select more appropriate fine-grained motor patterns. The ability to offload in this way is one of the features that makes a phenomenal interface consistently evolvable.

Second, the particular form of realization is important to distinguish organisms with the capacity for subjective experience from other things which might, at some very abstract computational level, do the things that we have attributed to a phenomenal interface. On the one hand, there is a close (and non-accidental) relationship between what a PI does and various forms of so-called deep reinforcement learning [87,88]. Yet extant forms of deep RL are, architecturally speaking, mostly feedforward. They also appear, from the point of view of processing nodes and computational expense, far less efficient than the recurrent biological solutions instantiated as π. We think that these architectures might end up mutually illuminating, but it is important to keep in mind that PIT is not committed to consciousness in any system that solves the problems of self-motion and action selection; it must also do so in the right way.

There is also a lower bound in the biological world: whatever slime moulds and pea plants are doing, it is unlikely that they are instantiating π. Similarly so with e.g. room-cleaning robots. That is true even if there is a way to describe what they are doing in a way that the phenomenal interface is described. That is because the relevant question is not the abstract description of what they do—*movement* and *self-location* and *world-mapping* and so forth—but the similarity between how they solve these problems and the transformation-function given by π.

So, for example, there is a sense in which *Caenorhabditis elegans* must integrate information: the motivational value of (say) food cues is downgraded after satiation, and different motivational drives mutually inhibit one another (see [89] for a good review). These processes largely operate in parallel with mutual inhibition, rather than transformation into a common evaluative framework as required by π. There is also no reason to think that such a setup would be capable of solving nl-MOMDPs in a meaningful way (parallel weighting implies a linear aggregation function; the absence of this is what makes nonlinear MOMDPs difficult [21,22]).

That said, it is important to emphasize that PIT is compatible with a range of possible positions, corresponding to the level of abstraction at which π is understood. The level of abstraction that includes the visually impaired will almost certainly cover other mammals as well. We claim that the insect central complex, while working with very different circuitry, also instantiates π. It is an open, and empirical, question whether any non-biological computational systems instantiate π. The right level of description of π will almost surely be an information-processing one, which means that PIT is not bound to biological systems. Conversely, it is not at all guaranteed that any extant systems instantiate a suitable PI, and it is not obvious that any plausible extensions of them would either [90].

## 5. Conclusion

PIT is a theory of the mechanisms of consciousness not a solution to the so-called ‘hard problem’ [91]. It fits with a standard empiricist line on the matter—a PI in humans ultimately performs a severe dimensionality reduction; hence, what we are aware of is much simpler than the processes that go into being aware [92,93]—but no more than that.

However, we think that PIT sheds light on a non-hard but extremely interesting problem, one that seems to have received relatively little attention in the literature on consciousness. Nagel pointed out that the conscious experience of a bat must be different to that of humans, in part because they have a wholly different modality of echolocation [6]. This is meant to make vivid the difficulties of conceiving what it is like to be a very different organism. Yet it also raises a more general issue: how it is that new properties might get added to phenomenal space at all as organisms evolve and complexify?

If one thinks that only humans are conscious, the problem does not arise. But once one thinks that consciousness can evolve by becoming richer (and the experience of a human is certainly richer than that of a fly), then there is a corresponding question about how the mechanisms of consciousness might support such a gradual expansion. This is, in some ways, to turn our original problem on its head. We started with the theoretical expansion of consciousness on behavioural grounds and asked about the mechanisms by which very simple organisms can end up conscious. Yet by focusing on simple organisms, we get a corresponding question of how it is that the richness of our own experience might have developed in evolutionary time.

One of the attractions of PIT is that it makes this problem salient in the course of offering a solution to it. Having a phenomenal interface means that body and cortex can expand, and the resulting system is still able to adjudicate between competing courses of action. PIT is not necessarily the only theory that has that property, but by emphasizing the *evolvability* of conscious experience, it opens a new direction for scientific study of consciousness.

## Data Availability

This article has no additional data.
